# Altered brain energetics induces mitochondrial fission arrest in Alzheimer’s Disease

**DOI:** 10.1038/srep18725

**Published:** 2016-01-05

**Authors:** Liang Zhang, Sergey Trushin, Trace A. Christensen, Benjamin V. Bachmeier, Benjamin Gateno, Andreas Schroeder, Jia Yao, Kie Itoh, Hiromi Sesaki, Wayne W. Poon, Karen H. Gylys, Emily R. Patterson, Joseph E. Parisi, Roberta Diaz Brinton, Jeffrey L. Salisbury, Eugenia Trushina

**Affiliations:** 1 Department of Neurology, Mayo Clinic, 200 First St. SW, Rochester, MN 55905; 2 Electron Microscopy Core Facility, Mayo Clinic, 200 First St. SW, Rochester, MN 55905; 3 Department of Pharmacology and Pharmaceutical Sciences, School of Pharmacy, University of Southern California, Los Angeles, CA, USA; 4 Department of Cell Biology, Johns Hopkins University School of Medicine, 725 N. Wolfe Street, Baltimore, MD, USA; 5 Institute for Memory Impairments and Neurological Disorders. University of California Irvine, CA, USA; 6 UCLA School of Nursing, CA, USA; 7 Laboratory Medicine and Pathology, Mayo Clinic, Rochester, MN, USA; 8 Neuroscience Program, University of Southern California, Los Angeles, CA, USA; 9 Department of Neurology, Keck School of Medicine, University of Southern California, Los Angeles, CA, USA; 10 Department of Pharmacology and Experimental Therapeutics, Mayo Clinic, 200 First St. SW, Rochester, MN 55905

## Abstract

Altered brain metabolism is associated with progression of Alzheimer’s Disease (AD). Mitochondria respond to bioenergetic changes by continuous fission and fusion. To account for three dimensional architecture of the brain tissue and organelles, we applied 3-dimensional electron microscopy (3D EM) reconstruction to visualize mitochondrial structure in the brain tissue from patients and mouse models of AD. We identified a previously unknown mitochondrial fission arrest phenotype that results in elongated interconnected organelles, “mitochondria-on-a-string” (MOAS). Our data suggest that MOAS formation may occur at the final stages of fission process and was not associated with altered translocation of activated dynamin related protein 1 (Drp1) to mitochondria but with reduced GTPase activity. Since MOAS formation was also observed in the brain tissue of wild-type mice in response to hypoxia or during chronological aging, fission arrest may represent fundamental compensatory adaptation to bioenergetic stress providing protection against mitophagy that may preserve residual mitochondrial function. The discovery of novel mitochondrial phenotype that occurs in the brain tissue in response to energetic stress accurately detected only using 3D EM reconstruction argues for a major role of mitochondrial dynamics in regulating neuronal survival.

Alzheimer’s disease (AD) is characterized by the deposition of extracellular amyloid (Aβ) plaques, intraneuronal neurofibrillary tangles comprised of hyperphosphorylated tau protein (pTau), synaptic loss, and neuronal cell death^1^. Significant hypometabolic changes detected early in AD patients using ^18^F-fluorodeoxyglucose positron emission tomography suggest that abnormal energy metabolism underlies disease etiology^2^. Robust energy production in neurons is essential for synaptic activity and neuronal survival. Recent studies demonstrated that energy production is dependent on the ability of mitochondria to undergo cycles of fission and fusion collectively termed “mitochondrial dynamics”^3^,^4^,^5^. Fission and fusion machinery depends on the fidelity of dynamin related protein 1 (Drp1), mitochondrial fission factor (Mff), mitochondrial fission protein 1 (Fis1), mitofusin-1 and mitofusin-2 (Mfn1, Mfn2), and optical atrophy 1 (Opa1) protein^3^,^6^,^7^,^8^,^9^,^10^. These proteins also regulate the assembly and stability of the respiratory chain supercomplexes inducing the remodeling of mitochondrial cristae and ultimately shaping mitochondrial morphology in response to the energetic demand of the cell^11^,^12^, which directly affects the development and maintenance of synapses^13^. Excessive mitochondrial division has been observed in cellular and animal models of familial AD (FAD), and in AD patients^14^. Thus, understanding regional responses to changes associated with disease progression, particularly regarding the relationship between mitochondrial energetics and the balance of mitochondrial fission and fusion, has the dual potential to elucidate basic mechanisms of disease and to suggest therapeutic targets. However, most of the studies conducted to date failed to account for three-dimensional architecture of the brain tissue and organelles presenting critical barrier to better understanding of mitochondrial dynamics in AD. Here, using three-dimensional electron microscopy (3D EM) reconstruction, we identified a novel mitochondrial fission arrest phenotype that might represent fundamental compensatory adaptation to bioenergetic stress, which is relevant but not limited to AD.

## Results

### Extensive MOAS formation in FAD animals

**Figure 1:**
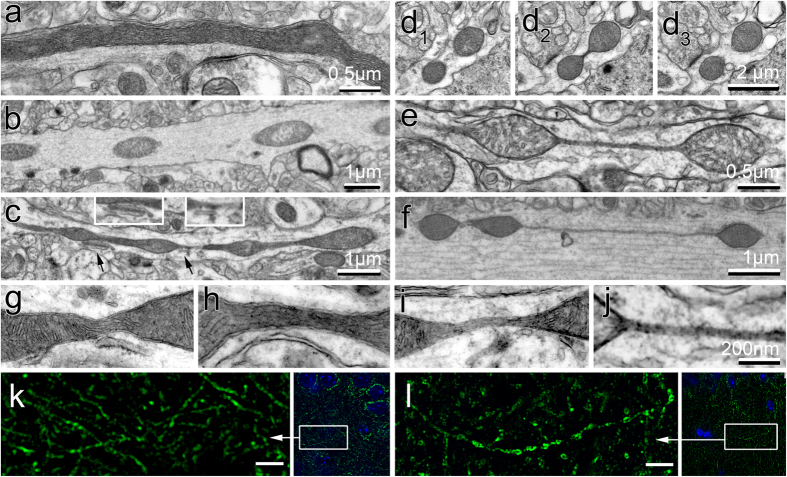
Mitochondrial morphology in CA1 hippocampi of NTG and FAD mice visualized using standard TEM and super-resolution immunofluorescence. (**a**) Mitochondrion in a neuropil in brain tissue of a NTG mouse. (**b**–**f**) Micrographs of mitochondrial profiles in the brain tissue of APP (**b**), 3xTgAD (**c**,**d**), and APP/PS1 (**e**,**f**) mice. (**d**) Consecutive serial sections of hippocampi from 3xTgAD mouse showing mitochondrial fission. (**g–j**) Membrane connections that contain mitochondrial matrix (**g,h**) or are devoid of matrix components (**i,j**) observed in APP/PS1 mice. (**k,l**) Mitochondria in brain tissue of NTG (**k**) or APP/PS1 (**l**) mice observed using Tom20 antibody (green) and a super-resolution fluorescence microscopy. Low magnification images on the right are co-stained with Hoechst (blue) to define nuclei. Scale bar, 2 μm. Three to five mice per each genotype were examined. All mice were females 40 weeks of age. Ten random sections with ~100 mitochondria were examined by blinded investigator for each brain tissue.

Using transmission electron microscopy (TEM), we examined mitochondria in the CA1 hippocampal region from 5 transgenic mouse models carrying human FAD mutations for presenilin 1 (PS1), amyloid precursor protein (APP), and mutant Tau protein (Table 1). Non-transgenic (NTG) littermates were used as control. Randomized blinded analysis of mitochondria in each brain tissue was restricted to neuropils longer than 3 μm representing axons. We found that compared to uniformly elongated mitochondria in the hippocampi of NTG mice (Fig. 1a), FAD mice exhibited a broader variety of mitochondrial shapes ranging from ovoid (0.3 by 0.5 μm in diameter, Fig. 1b) to teardrop profiles with tubular membrane extension(s) at one or both ends (Fig. 1c), and to teardrop shaped mitochondria (0.5 μm in diameter) connected by thin double membrane extending up to 5 μm long that we termed “mitochondria-on-a-string” (MOAS) (Fig. 1e,f). Fortuitous sections showed dividing mitochondria (0.3 μm in diameter) connected by short (~100 nm) membranes of uniform diameter (50–65 nm, Fig. 1d). This morphology became exaggerated in FAD animals where the double membrane MOAS connections varied in length and thickness, with broader connections containing mitochondrial matrix and cristae (Fig. 1c,g,h) and thin connections (uniformly ~65 nm in diameter) without matrix (Fig. 1e,f,i,j). We frequently observed apposition of MOAS and endoplasmic reticulum membranes at the junction between teardrop mitochondrial profiles and their connecting double membranes (Fig. 1c, inserts). MOAS were also observed in the brain tissue of APP/PS1 mice using super-resolution fluorescence microscopy (Fig. 1l).

**Figure 2:**
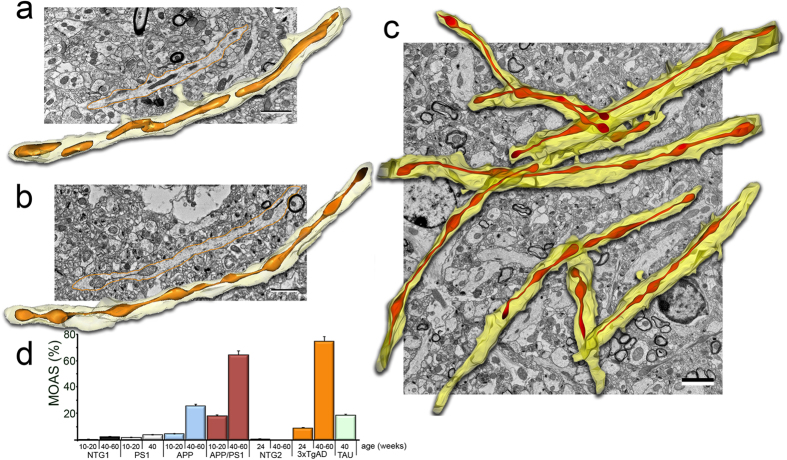
3D EM reconstruction of mitochondria in CA1 hippocampi of NTG and APP/PS1 mice. TEM images from ~28 serial sections 0.09 μm thick were stacked, aligned, and reconstructed using 3D reconstruction software in NTG (**a**) and APP/PS1 (**b,c**) mice 40 weeks of age (n = 3–5 per each group, all females). Scale bars, 2 μm. (**d**) Blind morphometric analysis of randomly selected EM of MOAS in CA1 brain region of FAD mice compared to NTG age-matched control at different ages (n = 3–9 mice per group, all females). Ten random sections with ~100 mitochondria were taken into analysis for each brain tissue. Data represent average ± SEM. NTG mice in the graph include NTG1 and NTG2 that did not have MOAS at 40 or 60 weeks of age.

The thickness of individual sections in TEM does not adequately represent the 3D architecture of mitochondria. This precludes critical analysis of the structure, which could predict possible functional failure. To address these issues, we examined mitochondrial morphology in hippocampi of FAD and NTG mice using 3D EM reconstruction of standard TEM images from 10–40 consecutive serial sections. We found an extensive MOAS phenotype in APP/PS1 mice compared to uniform, tubular morphology of mitochondria in NTG mice (Fig. 2a–c, Supplementary Movies 1–3). When larger regions of APP/PS1 hippocampi were imaged, MOAS were pervasive throughout most neuropils (Fig. 2c, Supplementary Movies 2 and 3). MOAS were most prevalent in APP/PS1 and 3xTgAD mice at 40 and 60 weeks of age reaching 50–70% (Fig. 2d) while almost no MOAS were found in age-matched NTG and PS1 mice at any age (Fig. 2d). MOAS were observed in ~20% of neuropils in Tau mice (Fig. 2d). These observations suggest that MOAS represent a novel phenotype prevalent in the brain tissues of FAD and Tau mice that increases in frequency with disease progression and accumulated mutations.

### MOAS are present in brain tissue of AD patients

**Figure 3:**
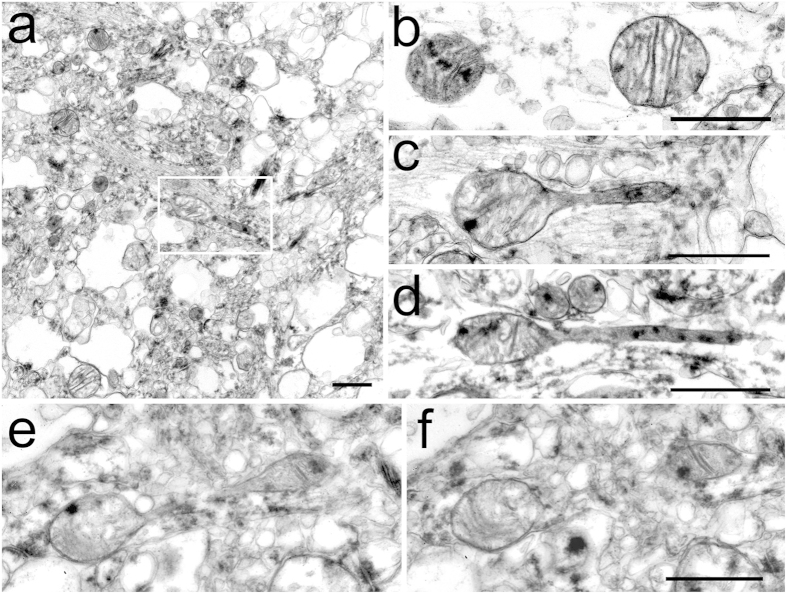
MOAS phenotype in hippocampi and entorhinal cortices of AD patients. The hippocampi, entorhinal cortices and cerebella were collected and fixed 6 – 24 hrs postmortem. (**a**) Low magnification representative survey image of hippocampus from an AD patient illustrating the overall degree of tissue preservation. The box contains teardrop shaped mitochondrion with membrane extension. (**b**) Representative EM micrograph of mitochondrial profiles in cerebellum from control individual. (**c,d**) Representative micrographs of teardrop shaped mitochondria with tubular membrane extensions observed in hippocampi from individuals diagnosed with AD (**c,d**). (**e,f**) Serial sections demonstrating MOAS phenotype in the hippocampus of an AD patient. Scale bars, 0.5 μm. Demographic data is presented in Supplementary Table S1.

We next examined whether MOAS formation is relevant to human disease. We assessed hippocampal, cortical, and cerebellar postmortem tissue (fixed within 5 to 24 hours after death, Supplementary Table S1) from cognitively normal individuals and AD patients. Consistent with rapid postmortem degradation, loss of tissue architecture, vacuolization of the cytoplasm and mitochondria dilatation were observed in all samples examined (Fig. 3a, Supplementary Fig. S1). No differences were found between neuronal mitochondria in the cerebella of control and AD patients where mitochondrial profiles were uniformly oval (~0.5 μm in diameter) with distinct cristae and occasional electron dense deposits (Fig. 3b). However, the examination of the CA1 hippocampal brain region, which is early and specifically affected in AD, revealed presence of teardrop mitochondria with tubular membrane extensions only in AD patients (Fig. 3a,c–e, Supplementary Fig. S1). These structures resembled MOAS observed in FAD mice (Fig. 1c). A MOAS phenotype in AD hippocampi was confirmed with serial sectioning (Fig. 3e,f). These data suggest that MOAS formation has relevance to a human disease condition.

### Enhanced Drp1 recruitment to MOAS in FAD mice

**Figure 4:**
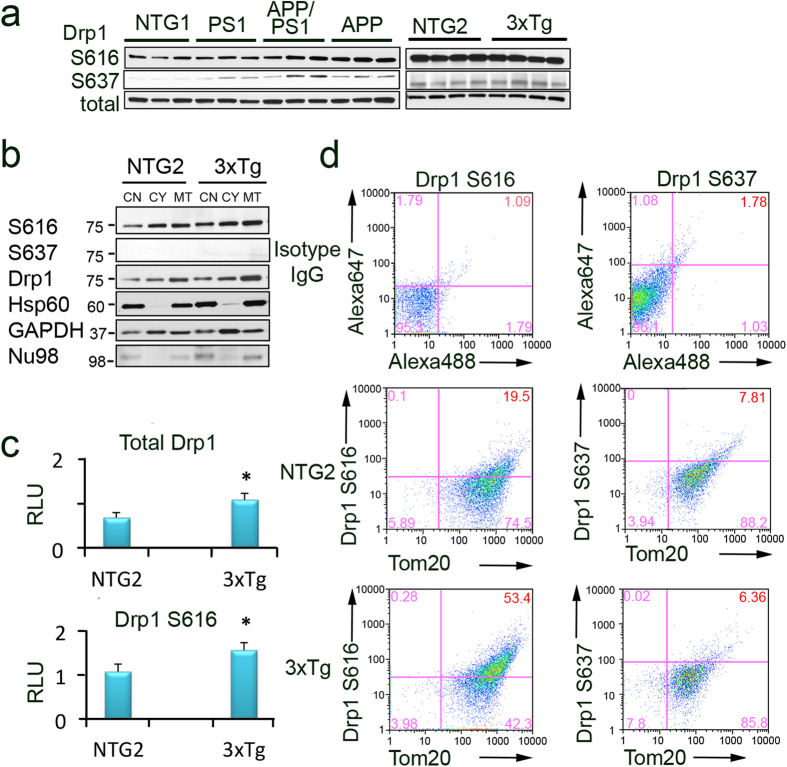
Activated Drp1 phosphorylated at S616 is localized to mitochondria in FAD animals. (**a**) Levels of Drp1 phosphorylated at S616 and S637 detected in the whole brain extracts from NTG1, APP, PS1, APP/PS1, NTG2 and 3xTgAD mice using Western blot analysis. Total Drp1 was used as loading control. Each lane represents individual mouse with three to four mice per group (all females, 40–60 weeks of age). Blots were not cropped. (**b**) Enhanced recruitment of Drp1 and Drp1 S616 to mitochondria isolated from hippocampi of 3xTgAD mice compared to age-matched NTG2 controls (female mice 60 weeks of age). CN – crude nuclear fraction; CY – cytoplasmic fraction; MT – mitochondria-enriched fraction. Heat shock protein 60 (Hsp60) confirmed mitochondrial enrichment and was used as loading control. Crude nuclear and cytoplasmic fractions were probed for nucleoporin 98 (Nu98) and glyceraldehyde 3-phosphate dehydrogenase (GAPDH). Cropped blots are represented; full-length blots are provided in Supplementary Fig. S3. (**c**) Densitometry analysis of Drp1 S616 and total Drp1 levels in mitochondrial fractions normalized to Hsp60 levels. Experiments were as in (**b**). Data represent average ± SEM of three independent experiments. RLU – relative light units. The asterisk denotes p < 0.05 in a paired sample Student’s t test versus NTG. (**d**) Recruitment of activated Drp1 phosphorylated at S616 to mitochondria increases in hippocampi of 3xTgAD mice compared to age-matched NTG2. Freshly isolated mitochondria from hippocampi (50 μg) were stained with either Drp1 S616 or Drp1 S637 antibodies together with Tom20 (mitochondrial marker) antibody. Mitochondria were gated based on light-scattering properties in the SSC and FSC modes and 20,000 events per sample were collected. To establish gating parameters, isotype non-specific IgG and the appropriate secondary antibodies conjugated with either Alexa 488 or 647 (top panels) were used. An increased phosphorylation of Drp 1 at S616 on mitochondria from AD (53%) vs. NTG (19.5%) animals indicates enhanced translocation (compare middle and bottom left panels). No changes in the level of Drp1 phosphorylated at S637 was found in mitochondrial fractions (compare middle and bottom right panels). Experiments were repeated three times.

To gain the insight into the molecular mechanism behind MOAS formation, we first evaluated the expression of key fission/fusion proteins in whole brain extracts and mitochondrial fractions from hippocampi of FAD and NTG mice (Fig. 4, Supplementary Figs S2 and S3). Overall expression level of Drp1, Mff, Fis1, Mfn1, Mfn2 or Opa1 in whole brain extracts from NTG and FAD mice was similar (Supplementary Figs S2 and S3). Likewise, no changes were noted in the abundance of the mitochondrial membrane protein Tom20 (Supplementary Figs S2 and S3). Thus, MOAS formation was not associated with a system-wide loss of fission/fusion proteins or significant changes in mitochondrial mass consistent with our previous observations^15^.

Mitochondrial fission is facilitated by phosphorylation of Drp1 at Serine position 616 (S616), which promotes its translocation to the mitochondria. Fission is inhibited by phosphorylation at S637, which releases Drp1 from mitochondria into the cytosol^8^. We found a significant increase in Drp1 S616 phosphorylation in whole brain extracts from APP/PS1 and APP mice (Fig. 4a, Supplementary Figs S2 and S3). However, no significant changes were detected between 3xTgAD and NTG2 animals (Fig. 4a, Supplementary Figs S2 and S3). Because whole brain extracts do not reflect mitochondria-associated proteins or brain regional differences, we next examined levels of Drp1 and its phosphorylation in enriched mitochondrial fractions isolated from the hippocampi of NTG and 3xTgAD mice using two complementary methods, Western blot analysis and flow cytometry (Fig. 4b–d, Supplementary Fig. S4). We found increased levels of Drp 1 phosphorylated at S616 in the enriched mitochondrial fractions from 3xTgAD mice (Fig. 4b,c), which was consistent with enhanced translocation of Drp1 to mitochondria detected in the whole brain extracts of APP/PS1 mice (Fig. 4a). As expected, the inactivated form of Drp1 S637 was not readily detected in mitochondria-enriched fractions (Fig. 4b).

In agreement with the results of Western blot analysis, increased levels of Drp 1 phosphorylated at S616 were observed on mitochondria from the 3xTgAD (53%) vs. NTG2 (19.5%) mice using flow cytometry and mitochondria-enriched fractions (Fig. 4d, Supplementary Fig. S4). Compared to Western blot analysis, application of flow cytometry allows evaluating proteins specifically associated with mitochondria since the analysis is restricted to the organelles by setting up the precise gating parameters (Supplementary Fig. S4). Thus, increased S616 phosphorylation of Drp1 and its translocation to hippocampal mitochondria was observed in animals with most pronounced MOAS phenotype.

### Hypoxia induces MOAS in WT mice

**Figure 5:**
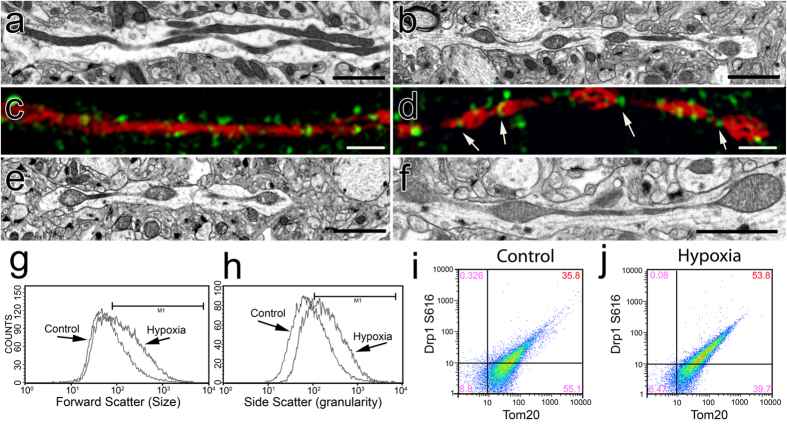
Acute hypoxia induces MOAS in WT mice. (**a,c**) Elongated mitochondria were observed using TEM (**a**) or super-resolution fluorescence microscopy with Tom20 (red) and Drp1 (green) antibodies (**c**) in the CA1 hippocampi of a WT mouse 10 weeks of age sacrificed by cervical dislocation without prior use of anesthetics. (**b,d**) Exposure of a WT mouse 10 weeks of age to CO_2_ for 5 min produced MOAS observed by TEM (**b**) or super-resolution fluorescence microscopy with Tom20 (red) and Drp1 (green) antibodies (**d**). (**e**) MOAS in the CA1 hippocampi in a WT mouse 88 weeks of age sacrificed by cervical dislocation. (**f**) Exposure of a WT mouse 88 weeks of age to CO_2_ for 5 min caused MOAS formation. Exposure of a WT mouse 10 weeks of age to CO_2_ for 5 min produced mitochondria with increased size (**g**, FSC histogram, median intensity of control 109.19 and hypoxia 203.0) and granularity (**h**, SSC histogram, median intensity of control 138.56 and hypoxia 268.44), and resulted in enhanced mitochondrial recruitment of Drp1 phosphorylated at S616 (53.8%, S616-Tom20 plots) in the CA1 hippocampal region compared to age-matched control. (**h–j**) Flow cytometry analysis of mitochondria isolated from hippocampi of mice exposed to hypoxic conditions compared to control mice (**g,h**) confirmed that enhanced recruitment of the activated Drp1 to mitochondria results in elongated organelles (**i,j**). Scale bars, 2 μm (**a,e,b,f**). Scale bars, 1 μm (**c,d**). Three to four female mice per each group were examined.

Since the recruitment and translocation of Drp1 to mitochondria was enhanced in FAD hippocampi, we next examined whether formation of MOAS was associated with bioenergetic changes characteristic of AD brain. Along with hypometabolism, chronic hypoxia and energetic stress are linked to the accumulation of Aβ in brain blood vessels in AD^16^. We therefore investigated whether hypoxia-induced stress promotes MOAS formation. We exposed wild type (WT) mice to acute hypoxic conditions using CO_2_. Age-matched WT mice euthanized by cervical dislocation without prior CO_2_ treatment were used as controls. EM evaluation revealed a pronounced formation of MOAS in ~80% of the neuropils examined in hippocampi of hypoxic young (10 weeks) WT mice compared to animals euthanized by cervical dislocation (Fig. 5a,b). Super-resolution fluorescence microscopy confirmed MOAS formation in hypoxic brain tissue of WT mice (Fig. 5c,d). Moreover, while Drp1 was discontinuously distributed along WT mitochondria (Fig. 5c), it formed rings localized to the regions of constriction along hypoxia-induced MOAS (Fig. 5d, arrows) suggesting normal Drp1 recruitment but incomplete fission. Older WT animals (88 weeks) euthanized using cervical dislocation had increased number of MOAS compared to younger WT animals regardless of hypoxia exposure (Fig. 5e,f) consistent with age-related decline in brain energetics. Flow cytometry analysis of mitochondria isolated from hippocampi of WT animals exposed to hypoxic conditions indicated increased size and granularity or internal complexity compared to control mice (Fig. 5g,h). This analysis also confirmed that formation of elongated organelles in these cases was associated with enhanced recruitment of the activated Drp1 phosphorylated at S616 to mitochondria (Fig. 5i,j). Collectively, these observations demonstrate that energy depletion induced by hypoxia^16^ or associated with chronological aging^17^ rather than aberrant Drp1 recruitment may contribute to MOAS formation.

### Inhibition of Drp1 induces MOAS formation

**Figure 6:**
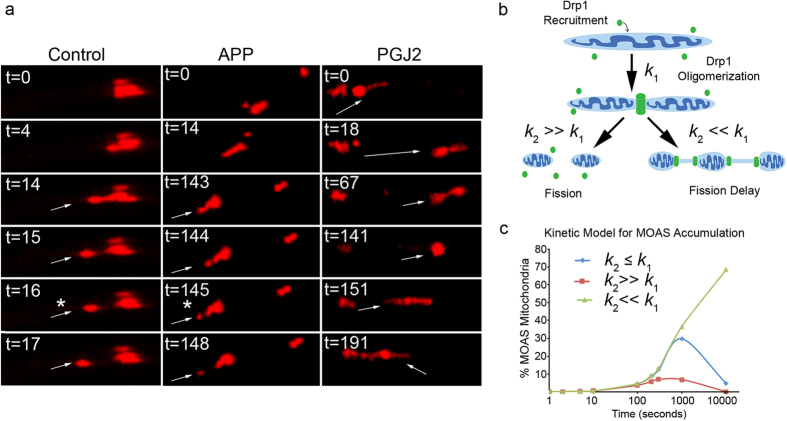
MOAS formation is associated with fission delay. (**a**) Real-time imaging (t, seconds) of mitochondrial axonal movement in live cortical neurons (E17) from WT (left panel), APP mice (middle panel), and WT neurons treated with 2 μM PGJ2 for 30 min prior to imaging (right panel). Arrows indicate progress along the axon of the same daughter mitochondrion produced after fission (asterisk) from parental organelle. Thirty individual mitochondria were examined from each of the three movies generated for each condition. Experiments were repeated in three independent platings. (**b**) Model of two-step reaction leading to fission arrest where the first step (*k*_1_) represents Drp1 recruitment to a mitochondrion and subsequent self-assembly resulting in formation of early fission intermediates. The second step (*k*_2_) consists of either a rapid GTP hydrolysis-driven constriction resulting in mitochondrial membrane scission (*k*_2_ ≫ *k*_1_) or a decrease in the membrane constriction/scission rate resulting in MOAS formation (*k*_2_ ≪ *k*_1_). (**c**) Kinetic model of MOAS accumulation as an intermediate product during the reaction 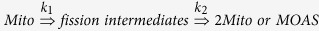 calculated as 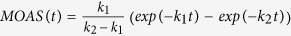 for three different scenarios. First: *k*_2_ ≪ *k*_1_ (green line) where 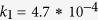 and 
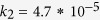
. Second: *k*_2_ ≫ *k*_1_ (red line) where 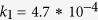 and 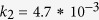. Third: 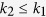 (blue line) where 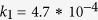 and 
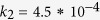
. Fission rate of mitochondria in cortical neurons used for calculations for 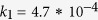 fission/mitochondria/s^−1^.

Since we found increased levels of activated Drp1 recruited to mitochondria in FAD mice and demonstrated that energy deprivation induces MOAS, we hypothesized that inhibition of Drp1 GTPase activity associated with energetic crisis may result in fission arrest or delay leading to formation of MOAS. To test this hypothesis, we investigated mitochondrial dynamics in live primary mouse cortical neurons using time-laps video microscopy (Fig. 6a). In WT untreated neurons, mitochondrial fission occurred rapidly, within ~15 seconds, following the earliest indication of MOAS formation (Fig. 6a Control, Supplementary Movie 4). In contrast, in neurons from FAD mice, mitochondria exhibited a marked fission delay (>2 minutes) (Fig. 6a APP, Supplementary Movie 5). To determine if inhibition of Drp1 GTPase activity is a rate-limiting step to persistence of MOAS, we treated WT neurons with physiological levels of 15-deoxy-Δ12,14-Prostaglandin J2 (PGJ2), a known GTPase inhibitor^18^, and investigated dynamics of mitochondrial fission. Treatment with PGJ2 did not affect mitochondrial movement, but generated persistence (>5 min) of the MOAS transition state (Fig. 6a PGJ2, Supplementary Movie 6). These observations suggest that inhibition of Drp1 GTPase activity could contribute to fission arrest and formation of MOAS in healthy neurons.

## Discussion

Using 3D EM reconstruction, we identified novel neuronal mitochondria phenotype that was present in animal models and in humans in response to energetic stress associated with AD, hypoxia and aging. Contrary to the previous reports, we did not detect excessive mitochondrial fragmentation in AD but rather observed MOAS phenotype that increased with disease progression and was more pronounced in animals with multiple FAD mutations. Mitochondrial fragmentation has been linked to the development of AD^14^,^19^ suggesting that strategies designed to normalize fission/fusion machinery may attenuate disease progression. Nevertheless, most of the methods utilized to study mitochondrial dynamics did not take into consideration significant complexity of the intact brain environment with preserved 3D architecture of the tissue and mitochondria. Application of 3D EM reconstruction in our study clearly identified novel mitochondrial phenotype that could otherwise be easily mistaken for multiple fragmented organelles if examined using conventional fluorescence microscopy or TEM. Moreover, even with the loss of tissue integrity, we identified MOAS in the postmortem human AD brain tissues primarily affected in the disease.

Mitochondrial fission is a multistep process, which includes Drp1 activation via S616 phosphorylation, Drp1 recruitment and self-assembly on mitochondria, GTP-driven constriction of mitochondrial outer membrane (MOM), final scission and membrane remodeling^20^. Failure at any step could lead to fission arrest and formation of step-specific fission intermediates. Our findings indicate that MOAS formation is not associated with altered levels of expression of fission/fusion proteins or with impaired Drp1 activation, recruitment or assembly on mitochondria. In support of our data, the inhibition of Drp1 recruitment by PKA-mediated negative regulatory phosphorylation at S637/S656^21^, prevention of self-assembly by mdivi-1 inhibitor^22^ or knockout of Drp1 expression^23^ leads to the formation of long interconnected organelles but not MOAS. Moreover, we found increased localization of Drp1 phosphorylated at S616 on mitochondria in FAD mice, which paradoxically produced MOAS rather than expected increase in fragmented mitochondria. In this regard, others also have shown that overexpression of mitochondrial Drp1 receptors MiD49/51^24^, expression of a dominant-negative mutant of MARCH5, a mitochondria-associated E3 ubiquitin ligase^25^ or down-regulation of F-actin regulatory proteins cortactin and cofilin^26^ prevented mitochondrial fission despite increased recruitment and proper assembly of Drp1 at scission sites. Our data suggest that MOAS are formed during the final steps of the fission process associated with Drp1 GTP hydrolysis and includes the formation of early (Fig. 1g,h) and late (Fig. 1i,j) intermediates. Examination of the width of Drp1 assembly-constricted mitochondrial membranes on early fission intermediates revealed a consistent diameter of ~160 nm in FAD, hypoxic and older WT mice (Fig. 5b,e,f, Supplementary Table S2). This is consistent with the diameter of the membranes associated with self-assembled Drp1 prior to constriction driven by GTP hydrolysis determined using lipid tubes (121 ± 25 nm)^27^ or isolated mitochondria (137 ± 60 nm)^28^. During GTP hydrolysis, Drp1-mediated fission further constricts MOMs producing final fission intermediates with membrane width reduced to ~80 nm^28^. The width of late fission intermediate membranes observed in our study falls in the same range: 67–94 nm in FAD and hypoxic WT mice (Supplementary Table S2). This is consistent with the hypothesis that MOAS represent fission intermediates formed at the GTP-driven hydrolysis constriction step before scission and membrane remodeling (Fig. 6b).

Analysis of the time-lapse images of mitochondrial dynamics demonstrated significant delay in fission in neurons from FAD mice (~15 fold) and WT neurons treated with PGJ2 (~30 fold) compared to untreated cells, which also supports our hypothesis that MOAS accumulation in the brain tissue of FAD mice is associated with the impairment of Drp1 function at the step of GTP hydrolysis. Kinetic modeling of fission where MOAS are considered as intermediate products of a simplified two-step reaction confirms MOAS accumulation within a few minutes if the efficiency of a final fission step is inhibited by 10-fold (Fig. 6b,c). Thus, our data argue that inhibition of Drp1 activity at the last step of fission is responsible for MOAS formation.

While we demonstrated that altered energetics, well documented in FAD mice^15^ and AD patients^2^, are sufficient to cause fission arrest, other mechanisms associated with AD could contribute independently or in a combination to MOAS formation. Thus, production of PGJ2 by activated microglia^29^ can reduce GTPase activity of Drp1. Indeed, others have shown that incomplete fission and formation of elongated mitochondria with the width of the scission membranes ~91 nm was observed under conditions where GTP concentration was reduced^27^. GTP deficiency also significantly decreased the rate of Drp1 dissociation from lipid membranes upon completed fission causing accumulation of membrane-bound Drp1^27^. Furthermore, MOAS formation can occur at the very last step of fission process, at the stage of scission and membrane remodeling. While membrane constriction driven by GTP hydrolysis occurs within hundreds of milliseconds, membrane remodeling associated with scission takes seconds to complete depending on the tension and membrane elasticity^30^. Membranes with high tension break within seconds^31^, while membranes with low tension require minutes^32^. Therefore, changes in mitochondrial membrane tension and elasticity due to altered lipid composition of MOMs^33^ could also contribute to MOAS formation. Within our samples, interconnected membranes in MOAS reached up to 5 μm in length while normal fission intermediates are typically no longer than 100 nm. It is feasible that low tension of the MOAS membranes could arise from decreased mitochondrial motility observed in FAD mice^15^ and AD patients^34^. Indeed, the most substantial MOAS accumulation was observed in 3xTgAD and APP/PS1 mice where axonal trafficking of mitochondria is significantly inhibited^15^,^35^. The interconnection of altered axonal trafficking, Drp1 activity and mitochondrial dynamics is further supported by the observations that mitochondrial axonal motility is specifically dependent on the levels of ATP produced by the organelles^36^. Thus, negative impact on the energetics could initiate a vicious cycle affecting Drp1 activity, mitochondrial dynamics, motility and function ultimately contributing to AD progression. Mitochondria in the hippocampi of PS1 or Tau mice did not exhibit significant amount of MOAS consistent with a lesser effect of these mutations on trafficking, and significantly later onset of disease phenotype^37^.

Finally, it is interesting to speculate on functional role of MOAS. Mitochondrial fission is essential for quality control ensuring the removal of damaged organelles via mitophagy^3^. It has been shown that highly fused mitochondria are formed in response to bioenergetic changes under conditions of nutrient deprivation or exposure to certain forms of stress^38^. Such rearrangement provides stress resistance and protects organelles against mitophagy^39^,^40^. Similar, the activity of Drp1 in 3xTgAD mice was inhibited during starvation leading to elongated mitochondria, which protected them from engulfment by the phagophore and subsequent degradation^39^,^40^. While the details of molecular mechanisms, especially with respect to the involvement of other fission/fusion proteins, remain to be determined, it is feasible that MOAS formation allows mitochondria to sustain cell viability during energy deprivation as in case of AD or hypoxia contributing residual functions towards extending a protective energetic margin that plays a role in neuronal survival. Taken together, our study reveals greater complexity of mitochondrial fission/fusion that includes sustained transition state dynamics. The occurrence of delayed fission during normal aging, hypoxia or neurodegenerative diseases provides compelling evidence for fundamental compensatory adaptation of mitochondria to bioenergetic stress.

## Methods

### Animals

The following mice were used in the study: APP_SWE_ (K670N, M671L)^41^; PS1 (M146L)^42^; double transgenic APP/PS1^43^; non-transgenic (NTG1) littermates for APP/PS1; triple transgenic mutant 3xTgAD mice^44^ overexpressing PS1(M146V), APP(Swe), and tau(P301L) (animals were bred and maintained by University of Southern California, tissue was provided by Dr. Brinton); non-transgenic littermates (NTG2); Tau mice harboring human tau transgene with P301S mutation^45^ (provided by Dr. D. Baker, Mayo Clinic Rochester, MN); and C57B6/J (WT) mice were used in hypoxia experiments (Table 1). The animals were genotyped for the expression of both transgenes by a PCR method using a sample of mouse-tail DNA. The characterization of amyloid and tau pathologies, as well as synaptic dysfunction in 3xTgAD mice has been described previously^44^ and confirmed in Dr. Brinton’s laboratory. Mitochondrial dysfunction in FAD and Tau mice that includes reduced mitochondrial membrane potential and ATP levels, decreased mitochondrial dynamics and hypometabolism was described previously^15^,^46^,^47^,^48^,^49^,^50^. Mice were genotyped routinely to confirm the purity of the colony. Mice were housed on 12 hours light/dark cycles and provided *ad libitum* access to food and water, with a regular feeding and cage-cleaning schedule. All procedures were performed using humane and ethical protocols approved by the Mayo Clinic and the University of Southern California Institutional Animal Care and Use Committees and in accordance with the National Institute of Health’s Guide for the Care and Use of Laboratory Animals. Three to nine animals were used per each group/experiment; all animals were females. Mice were matched by age and genotype, and were randomly assigned to the study group based on the age and genotype. The number of animals in each group was determined based on the 95% of chance to detect changes in 30–50% of animals. The following exclusion criteria were established: significant (15%) weight loss, changing in the grooming habits (hair loss), pronounced motor dysfunction (paralyses), or other visible signs of distress (unhealed wounds).

### Human brain tissues

Experiments with post-mortem human brain tissue were approved by the Mayo Clinic IRB (#12-007847) and were carried out in accordance with the approved guidelines. Informed consent was obtained from all subjects involved in the study. Eight postmortem brain specimens from AD patients and age-matched control subjects were obtained from the Mayo Clinic, UCLA and the UCI Tissue Repository. All patients were randomly assigned to the study group based on the disease diagnosis. Demographic details on each subject are presented in Supplementary Table S1. Six specimens were from patients diagnosed with AD according to Braak criteria, and two were from age-matched control subjects. The AD brain specimens were classified from Braak stages I and II (early AD), III and IV (definite AD), V and VI (severe AD) or control subjects based on quantitative pathological features including senile plaques, neurofibrillary tangles and neuronal density. One of the subjects also had tauopathy with mostly tangles. The specimens from the hippocampi, entorhinal cortices and cerebella were immediately fixed in Trump’s solution (4% formaldehyde + 0.1% glutaraldehyde in 0.1M phosphate buffer) for electron microscopy processing.

### Electron microscopy

EM reconstruction was done in three to five mice from each genotype (NTG1, APP, PS1, APP/PS1, NTG2, 3xTgAD, Tau). For TEM, mice were anesthetized with either intraperitoneal injection of ketamine/xylazine or inhalation of a mixture of 5% isoflurane and oxygen followed by cardio perfusion with 4% paraformaldehyde (PFA). Whole brains were removed and post-fixed in Trump’s solution (4% formaldehyde + 0.1% glutaraldehyde in 0.1M phosphate buffer) overnight at room temperature. The hippocampus (CA1 region) was dissected from each hemisphere and processed for TEM. Tissue was fixed in 1% osmium tetroxide and 1% aqueous uranyl acetate, dehydrated in a graded series of ethanol, and embedded in Embed 812/Araldite (EMS, Hatfield, PA). Thin sections (0.1 μm) were collected on copper grids, post-stained with lead citrate and viewed at 80 kV with a JEOL 1400 transmission electron microscope (JEOL USA, Peabody, MA). Ten random areas from each CA1 region were imaged, and only neuropils longer than 3 μm were selected for analyses. Mitochondrial profiles were scored according to their appearance as regular elongated (>3 μm long), ovoid (0.5 μm in diameter), teardrop shaped, or MOAS. Analyses were done by investigators blinded to the mouse genotype.

## 3D EM Reconstruction

For 3D EM reconstruction, thin (0.09 μm) serial sections (20–40 per mouse, n = 3 mice per genotype) were obtained from the blocks processed for conventional TEM. Serial sections were collected onto formvar coated slot grids (Pella, Redding CA), stained and imaged as described above. Images collected from 20–40 serial sections were then stacked, aligned, and visualized using *Reconstruct* software^51^. Reconstructions were performed on longitudinally sectioned neuropils that contained mitochondria.

### Mitochondrial fractionation

Fresh mouse brain tissue was washed with ice-cold PBS and immersed in 3 mL of mitochondrial isolation buffer (MIBA) containing 10 mM Tris-HCl, pH 7.4, 1 mM EDTA, 0.2 M D-mannitol, 0.05 M sucrose, 0.5 mM sodium orthovanadate, 1 mM sodium fluoride and 1× Complete protease inhibitor cocktail (Roche, USA). Tissue was homogenized using a Teflon pestle, and lysis was confirmed using light microscopy. The crude nuclei (CN) fraction was isolated from the lysate by centrifugation at 500 × *g* at 4 °C for 5 min. The remaining supernatant was centrifuged at 8000 × *g* for 10 min at 4 °C yielding heavy mitochondrial (MT) and cytoplasmic (CY) fractions. The MT pellet was washed three times with ice-cold MIBA buffer before it was resuspended in 1 mL of the same buffer. To assess organelle enrichment, 20 *μ*g of protein was separated using SDS-PAGE. After transfer to PVDF, immunoblot analysis was performed using GAPDH, HSP60 and Nu98 antibodies. Three mice from each experimental group were taken into analysis.

### Flow Cytometry

Flow cytometry analysis was done using FACSCanto digital flow cytometer equipped with a 488-nm Argon laser and a 635-nm laser (BD Biosciences, San Jose CA); FACSDiva software (BD Biosciences) was used for data collection. The final data analysis was performed using the FlowJo software (FlowJo, OR). To exclude debris, samples were gated based on light-scattering properties in the side-scattered light (SSC) and forward-scattered light (FSC) modes, and 20,000 events per sample within the gate were collected. The validity of gating was systematically confirmed by selective mitochondrial staining with 10-N-Nonyl acridine orange (NAO) (100 nM, Ex/Em: 488 nm/525 nm, Life Technologies, Grand Island NY), which binds cardiolipin in the inner mitochondrial membrane^52^, with 1,1,3,3,3,3-hexamethylindodicarbocyanine iodide (DiIC1(5)) (10 nM, Ex/Em: 633 nm/660 nm, Life Technologies) or with both stains. Briefly, 50 μg of mitochondrial fractions were stained at room temperature protected from light for 15 min in 400 μl of mitochondrial isolation MIBA buffer with 1× protease and 1× phosphatase inhibitors. The purity of mitochondrial fractions was consistently greater than 95% (Supplementary Fig. S3). To measure the level of Drp1 recruitment to mitochondria, enriched hippocampal mitochondria (50 μg) from either NTG2 or 3xTgAD mice were co-stained using the following antibody combinations: (1) Drp1 (1:50, BD Biosciences) and Tom20 (1:50, Santa Cruz Biotechnologies, Santa Cruz, CA); (2) Drp1 S616 (1:50, Cell Signaling, Boston, MA) and Tom 20; (3) Drp1 S637 (1:50 Cell Signaling) and Tom 20; or (4) isotype IgG (1:50, Thermo Scientific, Rockford, IL), which served as a staining control. The primary antibodies were incubated with the enriched mitochondria for 4 h in MIBA buffer at 4 °C. Secondary antibodies were labeled for 1 h under the same conditions following 3 washes using ice-cold MIBA buffer. Prior to FCM analysis, mitochondrial fractions were fixed using 4% PFA after final wash. Percent of Drp1, Drp1 S616 or Drp1 S637 localized on mitochondria was calculated when the particles showed double positive signals for Drp1/Tom20, Drp1 S616/Tom20 or Drp1 S637/Tom20. All experiments were performed in duplicates; 6 mice per each genotype were used for FCM experiments.

### Western Blot analysis

Whole brain extracts and enriched mitochondria fractions (20 μg of protein) obtained as described above were subjected to the separation by SDS-PAGE followed by immunoblot analysis using the following antibodies: DRP1 (1:400, BD Biosciences), DRP1 S616 (1:100, Cell Signaling), DRP1 S637 (1:100, Cell Signaling), OPA1 (1:400, BD Biosciences), HSP60 (1:200, Santa Cruz Biotechnologies), Nu98 (1:1000, Cell Signaling), GAPDH (1:1000, Cell Signaling), Mfn1 (1:500, Novus Biologicals, Littleton CO), Mfn2 (1:1000, Sigma, St Louis MO), Fis1 (1:500, Enzo Life Sciences, Farmingdale, NY), tubulin (1:5000, Sigma), MFF (1:1000, Abcam, San Francisco CA), and Tom20 (1:200, Santa Cruz Biotechnologies).

### Hypoxia experiments

Acute hypoxia was induced in C57B6/J (WT) mice 10 and 80 weeks of age (n = 3, all females) by exposure to CO_2_ using a 4-step preprogrammed chamber that controls the flow rate over 5 minutes and 10 seconds: (1) 2.5 L/min of CO_2_ for 30 sec; (2) wait for 40 sec; (3) 10 L/min of CO_2_ for 60 sec; (4) wait for 3 min. Immediately following the CO_2_ exposure, mice were cardio perfused using 4% PFA; and brains were removed and post-fixed in Trump’s solution overnight at room temperature. Control WT mice of the same age and sex were euthanized by cervical dislocation (CV). The CA1 hippocampal region was dissected from each hemisphere and processed for TEM as described above. For flow cytometry analysis, hippocampi were isolated from mice without cardio perfusion and immediately placed on ice. Enriched hippocampal mitochondria were isolated as described above.

### Immunohistochemistry

Mice were sacrificed as indicated in the corresponding figures, and brains were removed and fixed in 4% PFA for 2 hours at room temperature. Following cryoprotection in 30% sucrose, brains were embedded in optimal cutting temperature compound and coronally sectioned at 7 μm thickness on a cryostat. Slides were dried for 10 min at 37 °C, permeabilized for 5 min in −20 °C acetone and blocked for 30 min in solution containing 20% normal donkey serum and 0.1% BSA in PBS. Tom20 (1:200, Santa Cruz Biotechnologies) and total Drp1 (1:200, BD Bioscience) were visualized using indirect immunofluorescence (Alexa647 donkey anti rabbit, A31573, Alexa488, donkey anti mouse, A21202, both 1:500, Life Technologies). Hoechst was used to counterstain the nuclei (Hoechst 33342, Life Technologies). Sections were mounted using Prolong Gold anti-fade reagent (Life Technologies). Images were acquired with Zeiss Elyra PS.1 SR-SIM equipped with an Andor iXON897 EMCCD, alpha Plan-Apochromat 100x/1.46 Oil DIC M27 Elyra. Images were processed with Zeiss ZEN software black edition 2012.

### Neuronal cultures and time-lapse imaging

Primary cortical neurons were cultured as we described previously^15^. For time-lapse imaging, neurons from APP mice were isolated from individual cortices of newborn (P1) mice; genotyping was done prior to the day of experiment. Neurons from WT mice were isolated from cortices of embryonic (E17) mice. All experiments were performed in neurons cultured for 7 days unless specifically stated. Imaging of mitochondrial trafficking in live neurons was described previously^15^. Briefly, neurons were stained with TMRM (Molecular Probes, Eugene, OR); cells were maintained at 37 °C during imaging using confocal microscope LSM 510 (Carl Zeiss Inc, Germany) with a Plan-Apochromat 100 X (1.4 na) oil objective. Six hundred frames were collected in 10 min (one frame per second) and processed into a movie. The experiments were carried out using neurons from 3–5 independent platings. To inhibit Drp1 activity, WT neurons were treated with 2 μM 15-Δ-PGJ2 for 30 min prior to imaging.

### Statistical Analysis

Unless specifically mentioned, data were analyzed using Student t test. P < 0.01 was considered statistically significant.

## Additional Information

**How to cite this article**: Zhang, L. *et al.* Altered brain energetics induces mitochondrial fission arrest in Alzheimer’s Disease. *Sci. Rep.* **6**, 18725; doi: 10.1038/srep18725 (2016).

## Supplementary Material

Supplementary Information

Supplementary Movie S1

Supplementary Movie S2

Supplementary Movie S3

Supplementary Movie S4

Supplementary Movie S5

Supplementary Movie S6

## Figures and Tables

**Table 1:** Mouse models utilized in the study.

Mouse genotype	Abbrev.	Age, weeks	Pathology/ Mitochondrial alterations	Ref.
Human **APP** (K670N, M671L) under the control of the hamster prion protein promoter	APP	10–20; 40–60	Aβ plaques by 44 weeks; oxidative lipid damage, astrogliosis and microgliosis. No tangles. Impaired cognitive function at 24–40 weeks. Reduced mitochondrial membrane potential, ATP, altered dynamics and hypometabolism detected before the onset of amyloid plaques. MOAS are observed at 10 weeks	14; 15; 41; 46; 48; 49
Human **PSEN1** (M146L) driven by the rat PDGF-β promoter	PS1	10–20; 40	No abnormal pathology up to 2.5 years. Elevated Aβ42. Cognitive impairment after 48 weeks; Altered mitochondrial motility detected in embryonic neurons; loss of mitochondrial activity and altered brain energetics detected at 32 weeks. Low MOAS levels	42; 15
Human **APP** (K670N, M671L) crossed with **PSEN1** (M146L). The two trans-genes segregate independently	APP/PS1	10–20; 40–60	Aβ accumulation at ~24 weeks. Dystrophic neurites and GFAP-positive astrocytes at ~24 weeks with later microglial activation. Progressive cognitive impairment starting at 24 weeks.Altered mitochondrial motility in embryonic neurons. Reduced mitochondrial membrane potential, ATP levels, altered dynamics and hypometabolism are detected before the onset of amyloid plaques. Substantial MOAS formation at 10 weeks	15; 43; 46
Human **PSEN1** (PS1M146V), **APP** (K670N, M671L) and **MAPT**(P30IL) transgenes integrated at a single locus under the control of the mouse Thy1.2 promoter	3xTgAD	24; 40–60	Age-related, progressive neuropathology including plaques and tangles. Extracellular Aβ deposits by 24 weeks. Extensive Aβ deposits and Tau pathology by 48 weeks. Synaptic dysfunction, including LTP deficits, prior to plaques and tangles. Cognitive impairment by 16 weeks. Decreased mitochondrial respiration and pyruvate dehydrogenase protein level at 12 weeks. Increased hydrogen peroxide production and lipid peroxidation. Embryonic neurons exhibited significantly decreased mitochondrial respiration and increased glycolysis. MOAS formation at 24 weeks	35; 44; 47
Human **Tau** (P301S) under the direction of the mouse prion protein promoter.	Tau	40	Neuron loss, brain atrophy and neurofibrillary tangles by 32 weeks. Neuroinflammation with microgliosis and astrocytosis. Impairments in spatial memory and learning ability in Morris water maze. About 80 percent mortality by 12 months. Presence of mitochondrial dysfunction starting at 28 weeks: dysregulated mitochondrial enzymes; the reduction of complex V, ATP levels, and NADH-ubiquinone oxidoreductase activities. MOAS observed at 40 weeks	45; 50

APP: amyloid precursor protein; PSEN1: presenilins 1; PDGF: platelet-derived growth factor; MAPT: microtubule-associated protein tau; GFAP: glial fibrillary acidic protein; LTP: long-term potentiation.
