# Supplementary material for: Altered brain energetics induces mitochondrial fission arrest in Alzheimer’s Disease
Source: Sci Rep. 2016 Jan 5;6:18725. doi: 10.1038/srep18725 (PMC4700525; doi:10.1038/srep18725)
Supplement: Supplementary Information [file srep18725-s1.pdf]

Altered brain energetics induces mitochondrial fission arrest in Alzheimer's Disease

Liang Zhang[1,#], Sergey Trushin[1,#], Trace A. Christensen[2,#], Benjamin V. Bachmeier[1], Benjamin Gateno[1], Andreas Schroeder[1], Jia Yao[3], Kie Itoh[4], Hiromi Sesaki[4], Wayne W. Poon[5], Karen H. Gylys[6], Emily R. Patterson[7], Joseph E. Parisi[7], Roberta Diaz Brinton[3,8,9], Jeffrey L. Salisbury[2,10], and Eugenia Trushina[1,11,*]

Supplementary Figure S1. Mitochondrial phenotype in hippocampus and entorhinal cortex of control individuals. The hippocampi and entorhinal cortices were collected and fixed 6 – 24 hrs postmortem. (a) Representative EM micrograph of hippocampus from control individual. Scale bars, 2 μm. (b) Representative EM micrograph of entorhinal cortex from control individual. Scale bars, 1 μm. Demographic data is presented in Supplementary Table S1.

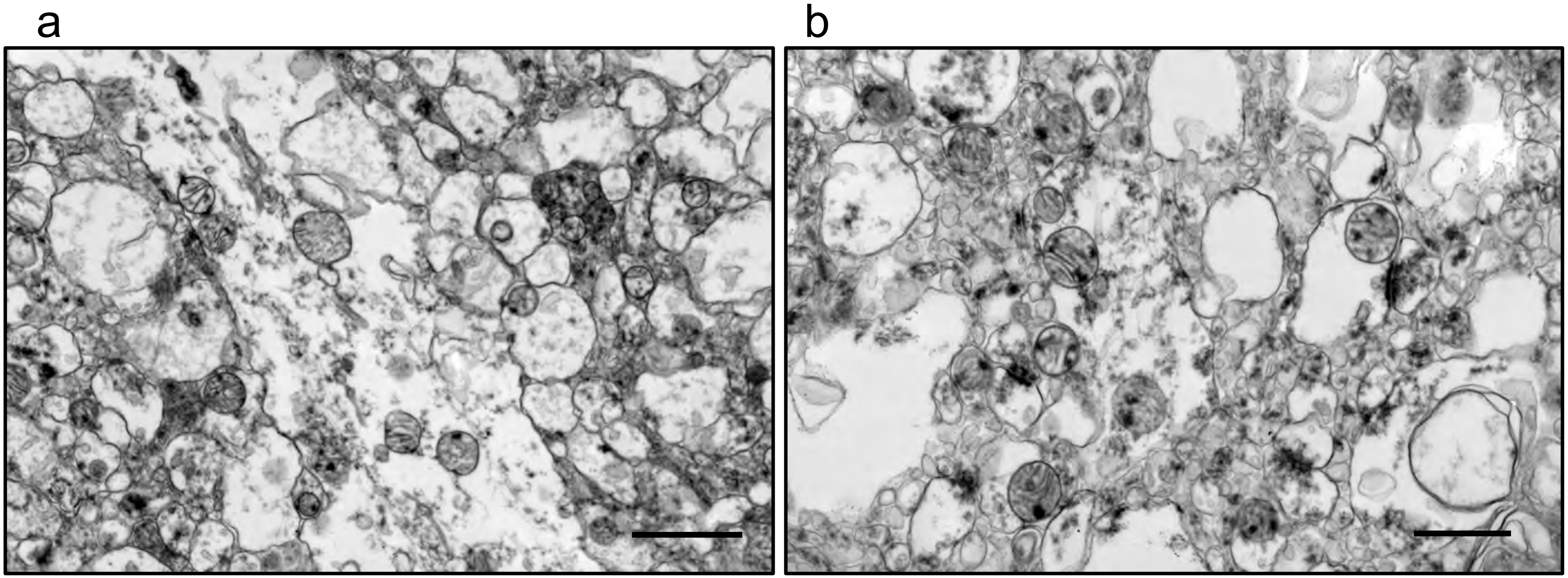

Supplementary Fig. S2. Expression of fission/fusion proteins in the brain tissue of FAD mice.

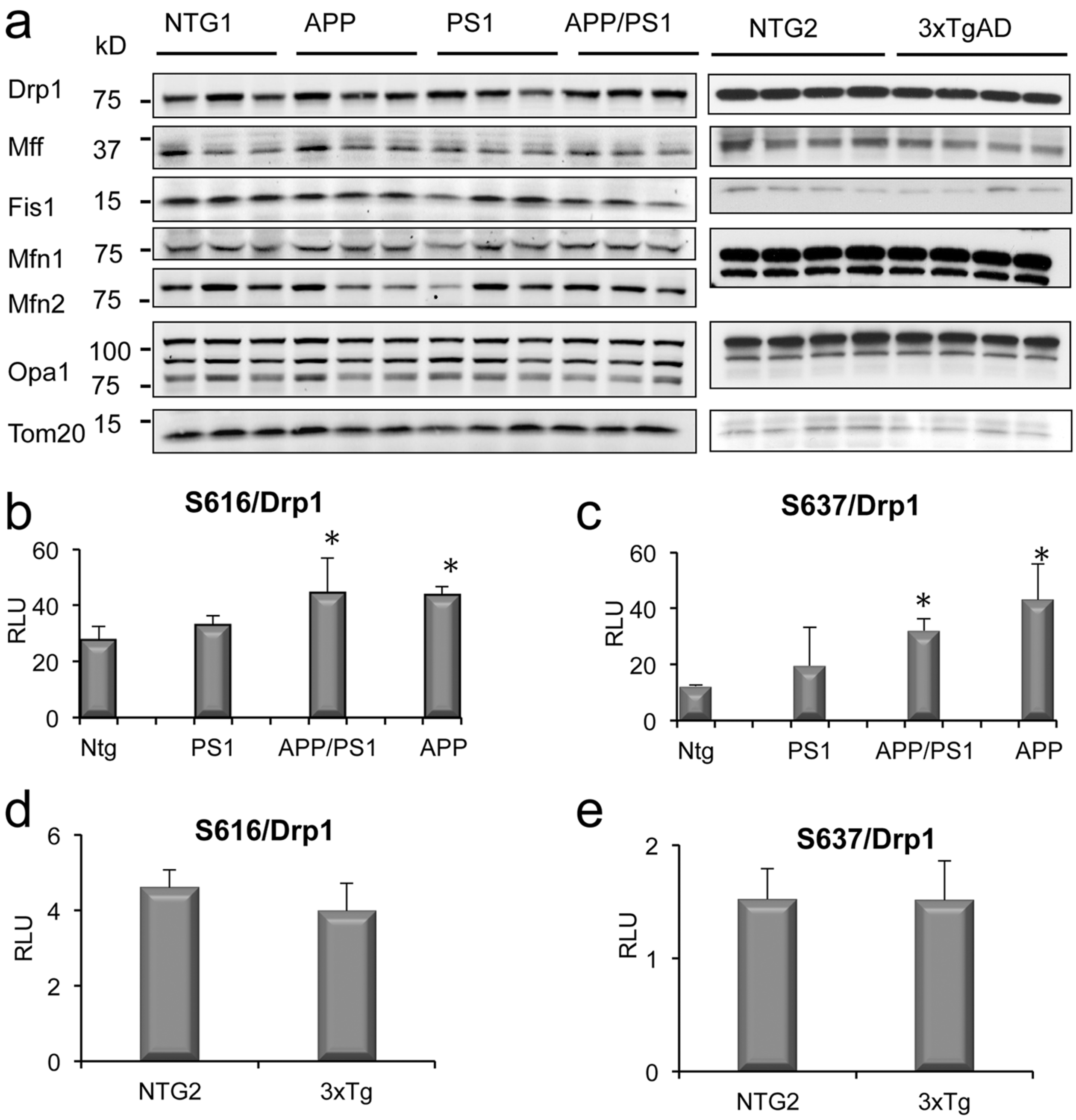

Supplementary Figure S3. Full blots of the Figure 4b

NTG2 3xTgAD NTG2 3xTgAD

CN LM HM CN LM HM CN LM HM CN LM HM

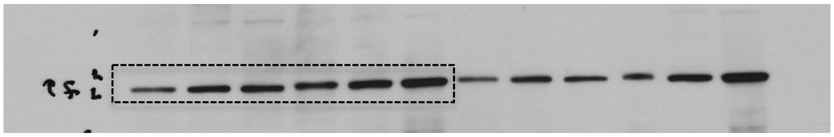

Drp1

NTG2 3xTgAD NTG2 3xTgAD

CN LM HM CN LM HM CN LM HM CN LM HM

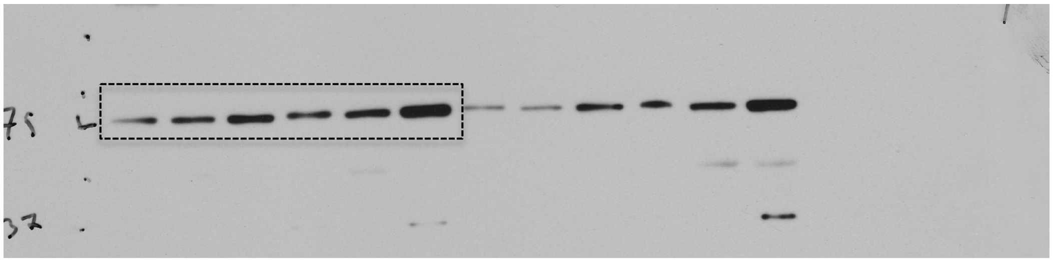

S637 Drp1

NTG2 3xTgAD NTG2 3xTgAD

CN LM HM CN LM HM CN LM HM CN LM HM

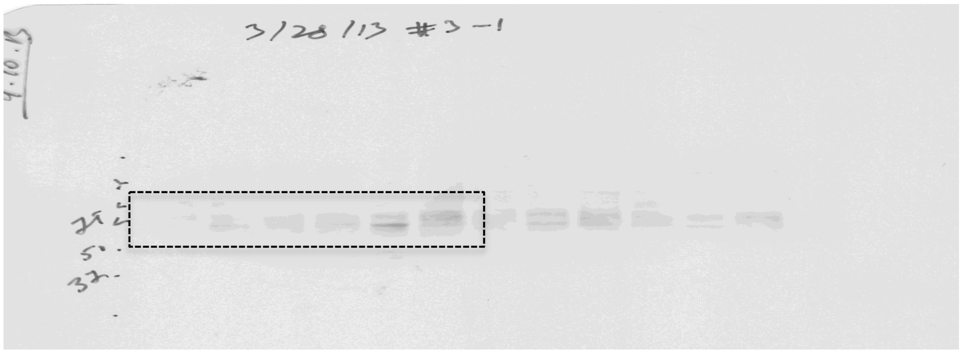

Supplementary Figure S3: Full blots of the Figure 4b (continuation)

NTG2 3xTgAD NTG2 3xTgAD

CN LM HM CN LM HM CN LM HM CN LM HM

GAPDH

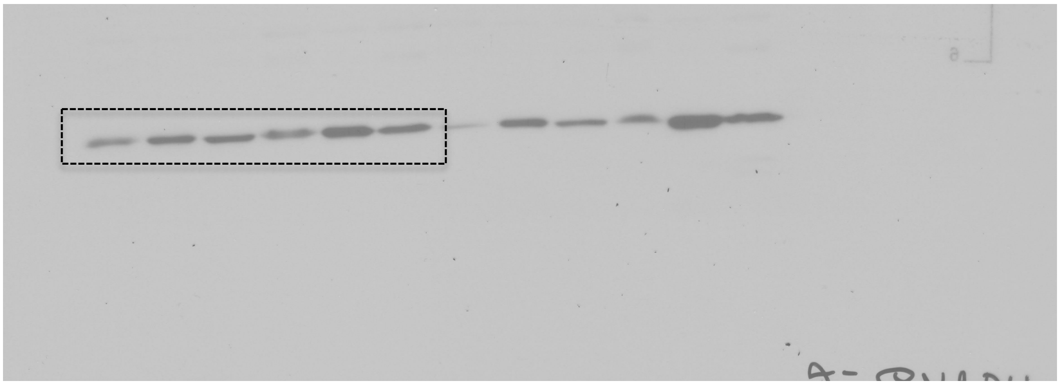

Hsp60

NTG2 3xTgAD NTG2 3xTgAD

CN LM HM CN LM HM CN LM HM CN LM HM

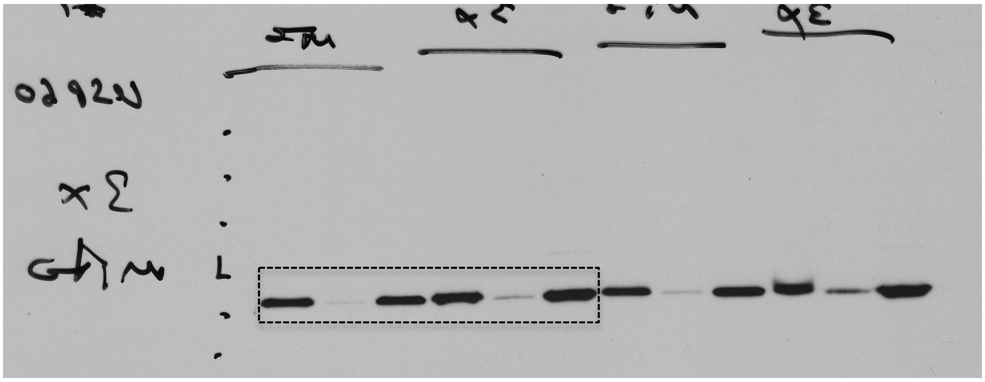

Nu98

NTG2 3xTgAD NTG2 3xTgAD

CN LM HM CN LM HM CN LM HM CN LM HM

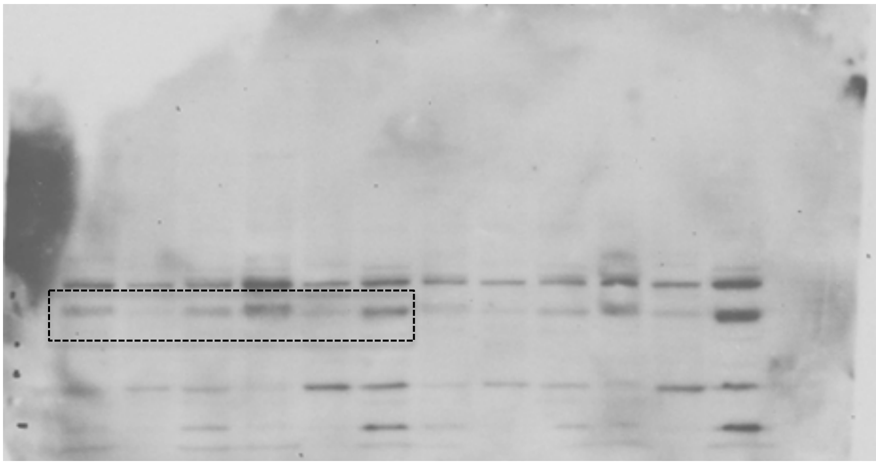

Supplementary Figure S4. Purity of mitochondrial fractions estimated using FACS. Freshly isolated mitochondria from the whole brain tissue of a WT mouse (50 mg) were stained with either NAO, DiIC1(5) or both. Mitochondria were gated based on light-scattering properties in the SSC and FSC modes (R1). 20,000 events per sample within the gate R1 were collected.

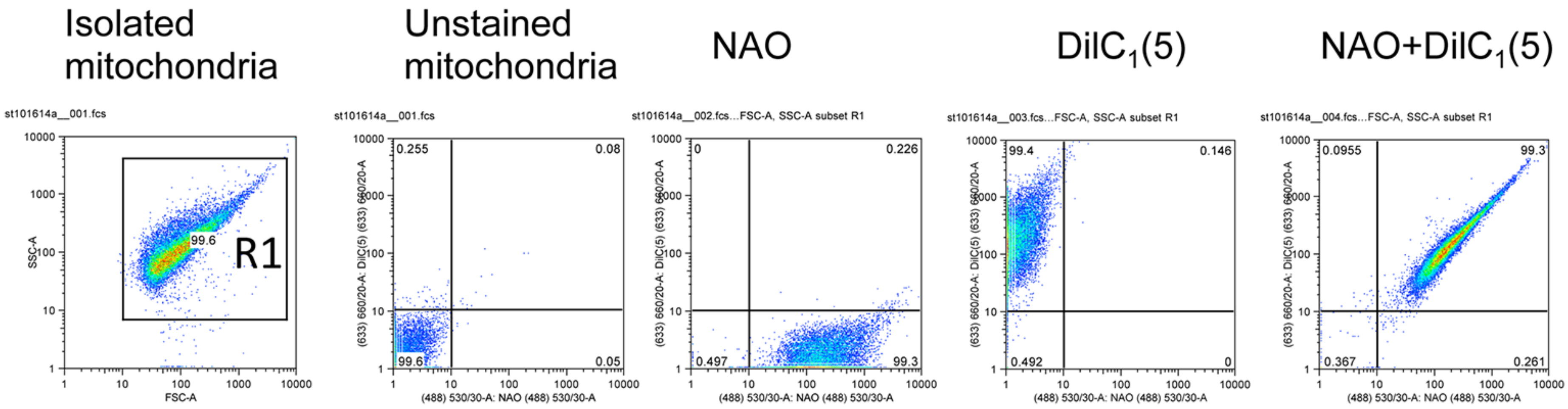

Supplementary Table S1. Demographic data on postmortem brain specimens utilized in the study

Case Type	Age/Sex	Postmortem interval, hrs	Braak stage
Control[1]	98/F	24	0
Control[3]	99/F	6	0
AD[1]	87/F	18	IV/V
AD[1]	86/F	8	III/IV
AD[2]	98/F	6	V
AD[3]	71/M	7	VI
AD[3]	74/M	6	VI
AD[2, 4]	85/F	5	VI

[1] Sample was obtained from Mayo Clinic Rochester

[2] Sample was obtained from UCLA Tissue Repository

[3] Sample was obtained from UCI Tissue Repository

[4] This patient also had a tauopathy with mostly tangles.

Supplementary Table S2. Width of MOAS membranes in NTG, APP/PS1 and 3xTg mice and young and old hypoxic WT mice.

Genotype	Early fission intermediates[1]	Late fission intermediates [2]
	Width[3], nm	Width[3], nm
NTG[4]	179 ± 50	0[7]
APP/PS1[4]	145 ± 36	84 ± 22
3xTg[4]	161 ± 22	94 ± 22
Young hypoxic[5]	164 ± 33	83 ± 15
Old hypoxic[6]	144 ± 23	67 ± 17

[1] Early fission intermediates contain mitochondrial matrix and cristae (Figure 1c,g,h)

[2] Late fission intermediates have long thin connections without matrix and internal membrane structures (Figure 1e,f,i,j)

[3] One hundred mitochondria profiles obtained with TEM similar to presented in Figure 1 were used in the analysis for each genotype or treatment.

[4] Data was generated in three female mice 40 weeks of age

[5] Data was generated in three female mice 10 weeks of age

[6] Data was generated in three female mice 88 weeks of age

[7] No MOAS were observed in NTG mice 40 weeks of age.

Supplementary Figure Legends

Supplementary Figure S1. Mitochondrial phenotype in hippocampus and entorhinal cortex of control individuals. The hippocampi and entorhinal cortices were collected and fixed 6 – 24 hrs postmortem. (a) Representative EM micrograph of hippocampus from control individual. Scale bars, 2 μm. (b) Representative EM micrograph of entorhinal cortex from control individual. Scale bars, 1 μm. Demographic data is presented in Supplementary Table S1.

Supplementary Figure S2. Expression of fission/fusion proteins in the brain tissue of FAD mice. (a) Representative western blot of the whole brain extracts from NTG, APP, PS1, APP/PS1 and 3xTgAD mice showing no significant changes in the levels of expression of key fission/fusion proteins. NTG1 are littermates for APP, PS1 and APP/PS1 mice; NTG2 are littermates for 3xTgAD mice. Tom20 was used as loading control. Each lane represents individual mouse with three mice per group. (b,c) Quantification of levels of Drp1 phopshorylated at S616 (b) and S637 (c) from Fig. 4a normalized to total Drp1 using densitometry. $^*P < 0.05$. $n = 3$ mice per group. (d,e) Quantification of levels of Drp1 phosphorylated at S616 (d) and S637 (e) in whole brain extracts from 3xTgAD mice in Fig. 4a normalized to total Drp1 using densitometry. $N = 4$ mice per group.

Supplementary Figure S3. Original full-scale blots from Figure 4b.

Supplementary Figure S4. Purity of mitochondrial fractions estimated using FACS. Freshly isolated mitochondria from the whole brain tissue of a WT mouse (50 mg) were stained with either NAO, DiIC1(5) or both. Mitochondria were gated based on light-scattering properties in the SSC and FSC modes (R1). 20,000 events per sample within the gate R1 were collected.

Supplementary Movie S1. 3D EM reconstruction of individual neuropils from the hippocampi of NTG (left) and APP/PS1 (right) mice 40 weeks of age. Two representative sets of standard TEM images from 10 - 40 consecutive serial sections 0.09 um thick show uniform, tubular morphology for mitochondria in a NTG mouse (left) compared to extensive MOAS phenotype in APP/PS1 mouse (right).

Supplementary Movie S2. 3D EM reconstruction of mitochondria in multiple neuropils in the hippocampi of NTG (left) and APP/PS1 (right) mice 40 weeks of age. Two representative sets of standard TEM images 0.09 um thick from 10 - 40 consecutive serial sections were taken into reconstruction.

Supplementary Movie S3. Magnified view of the MOAS in the hippocampi of APP/PS1 mouse 40 weeks of age. Representative set of standard TEM images from 40 consecutive serial sections 0.09 um thick were taken into reconstruction.

Supplementary Movie S4. Axonal motility in cortical neurons isolated from WT embryonic (E17) mice 7 days in culture. Cell body located at the top.

Supplementary Movie S5. Axonal motility in cortical neurons isolated from APP newborn (P1) pups 7 days in culture. Cell body located at the top.

Supplementary Movie S6. Axonal motility in cortical neurons isolated from WT embryonic (E17) mice 7 days in culture. Neurons were treated with 2 μM PGJ2 for 30 min prior to imaging. Cell body located at the top.
